# Long-term Outcomes of Knee Brace Versus Operative Medial Patellofemoral Ligament Repair in Children With First-Time Traumatic Patellar Dislocation: A 10-Year Follow-up From Skeletal Immaturity to Adulthood

**DOI:** 10.1177/23259671261432589

**Published:** 2026-04-27

**Authors:** Johan von Heideken, Elizabeth Arendt, Marie Askenberger

**Affiliations:** * Department of Women's and Children's Health, Karolinska Institutet, Karolinska University Hospital, Stockholm, Sweden; † Highly Specialized Pediatric Orthopedics and Pediatric Medicine, Astrid Lindgren Children's Hospital, Karolinska University Hospital, Stockholm, Sweden; ‡ Department of Orthopedic Surgery, University of Minnesota, Minneapolis, Minnesota, USA; Investigation performed at the Department of Women's and Children's Health, Karolinska Institutet, Karolinska University Hospital, Stockholm, Sweden

**Keywords:** patellar dislocation, MPFL repair, knee brace, pediatric, long-term follow-up, knee function

## Abstract

**Background:**

Optimal management of first-time traumatic patellar dislocation (FTPD) in pediatric patients remains disputed. Nonoperative treatment avoids surgical risks and may be optimal for select patients, whereas operative medial patellofemoral ligament (MPFL) repair aims to prevent recurrent instability and reduce redislocation in the short term.

**Purpose:**

To compare the redislocation rate, subsequent surgical intervention, and subjective knee function between pediatric patients initially treated with a knee brace (KB) and those who underwent operative MPFL repair.

**Study Design:**

Cohort study; Level of evidence, 2.

**Methods:**

This 10-year follow-up study of 74 participants from a previously conducted randomized controlled trial included 46 patients (62%) available for reassessment (KB, n = 25; MPFL repair, n = 21). The primary outcomes, redislocation and subsequent knee surgery, were collected from questionnaires and medical records. Subjective knee function was measured using the Knee injury and Osteoarthritis Outcome Score for children (KOOS-Child), Kujala Anterior Knee Pain Scale, and Tegner Activity Score. Key anatomic risk factors were taken from magnetic resonance imaging scans at baseline. Group comparisons were performed at 10-year follow-up.

**Results:**

The KB group demonstrated a higher overall redislocation rate (80%) compared with the MPFL repair group (62%), although the difference was not statistically significant (*P* = .175). Early redislocations (≤2 years) were more frequent in the KB group (52% vs 29%; *P* = .108), whereas rates for late (>2 years) redislocations (28% vs 33%) were similar. Among patients with redislocation, 45% (KB) and 54% (MPFL repair) underwent subsequent knee surgery. Surgery occurred earlier in the KB group (a mean of 25 vs 62 months). Long-term functional scores were similar between groups. However, patients who sustained any redislocation reported significantly lower KOOS-Pain, KOOS-Quality of Life, and Kujala scores than those who remained stable. Anatomic patellar instability factors were common in both groups.

**Conclusion:**

The long-term redislocation rate in FTPD for patients with MPFL repair was nearly as high as for patients treated nonoperatively. Subjective knee function was comparable between the 2 treatments. Given the high rate of recurring instability in children, the authors do not support routine MPFL repair for FTPD, nor do they support nonoperative treatment as the gold standard for every child. However, when nonoperative treatment is used, a structured follow-up should be mandatory.

Lateral patellar dislocation is a common acute knee injury in children and adolescents, with an estimated incidence of 60 to 150 per 100,000 person-years.^3,24^ Redislocation rates in this age group have been reported at 30% to 70%.^1,2,11,20^ Affected individuals often experience instability, pain, swelling, functional and psychological limitations, and reduced participation in sports.^6^ These findings underscore the importance of effective management to prevent long-term consequences.

When first-time traumatic patellar dislocation (FTPD) occurs without significant osteochondral or chondral lesions (≥1 cm^2^), the traditional approach has been nonoperative treatment, using a soft lateral patellar stabilizing brace for a short period followed by structured rehabilitation.^26^ Advocates of early surgery argue that stabilizing the patellofemoral joint may reduce the risk of further instability and improve knee-related quality of life, particularly in individuals at risk for instability due to anatomic factors and high levels of physical activity.^4,28^ However, surgery also carries risks such as persistent instability, patellofemoral pain, and reduced range of motion.^6^ Medial patellofemoral ligament (MPFL) reconstruction is increasingly favored over MPFL repair due to the superior midterm outcomes of reconstruction in terms of joint stability and redislocation prevention, particularly when combined with correction of relevant anatomic risk factors and treatment of osteochondral injuries, when indicated.^9,12,13,16^ Some orthopaedic surgeons still use MPFL repair as their main treatment for insufficient medial stabilizers.

In our previously published 2-year randomized trial of this cohort, arthroscopic MPFL repair reduced redislocation compared with a knee brace (KB); the difference was not sufficient for us to continue recommending the treatment because subjective and objective knee function did not statistically differ between groups.^2^

Several studies report short- to midterm (2-5 years) outcomes after FTPD in skeletally immature patients, but long-term comparative outcomes for both operative and nonoperative treatment are limited.^26^ Therefore, we performed a 10-year follow-up of this cohort regarding redislocation, subsequent surgery, and patient-reported knee function between the 2 treatment strategies.

## Methods

### Study Design and Participants

This follow-up builds on a previously published randomized controlled trial (RCT)^2^ that compared lateral patellar stabilizing soft brace (KB) treatment with arthroscopic-assisted MPFL repair after magnetic resonance imaging (MRI) verified FTPD without a significant osteochondral lesion ≥1 cm^2^. A total of 74 skeletally immature patients (aged 9-14 years) were enrolled initially between December 2009 and April 2012.

All 74 patients were contacted by mail, but 28 either declined participation or did not respond. At a mean follow-up of 10 years (range, 9-12 years), 46 (62%) patients were available for reassessment (KB group, n = 25; MPFL repair group, n = 21). Written informed consent was obtained from all participants, and the regional ethics board approved the study protocol (Dnr No. 2019-05869).

### Randomization, Crossover, and Treatment Protocols (Original Trial)

In the original trial, all patients underwent diagnostic arthroscopy irrespective of later allocation, and randomization to arthroscopic-assisted MPFL repair or no repair plus KB was performed intraoperatively. In this article, the term *nonoperative* refers to KB and does not exclude diagnostic arthroscopy. All analyses were performed using an intention-to-treat approach, retaining the 6 brace-allocated patients who crossed over to surgery during the first study period in the nonoperative group at the 2-year and 10-year follow-up assessments. At the 2-year follow-up, outcomes for the 6 patients who crossed over to surgery were presented separately. In the present 10-year follow-up, results from all patients in both original groups are included in the analyses.

— KB group: Patients received a lateral patellar stabilizing soft KB (Breg) that permitted full weightbearing and range of motion for 4 weeks.^2^
— MPFL repair group: Patients underwent arthroscopic MPFL repair with suture anchors placed at the patellar insertion site; femoral-side fixation was used only in 1 patient due to the injury. Postoperatively, a soft-cast splint allowed full weightbearing for 4 weeks.^2^
— Patients in both groups received a home training program and were referred to physical therapists with specialized knowledge regarding pediatric patellofemoral rehabilitation. The rehabilitation program focused on quadriceps and gluteal strengthening, range of motion exercises, gait retraining, and neuromuscular control.^2^

### Data Collection at 10-Year Follow-up

Participants who consented to participate in the follow-up received the same patient-reported outcome measures (PROMs) as in the first study: the Knee injury and Osteoarthritis Outcome Score for children (KOOS-Child),^19^ Kujala Anterior Knee Pain Scale,^14^ and Tegner Activity Score.^27^ Participants also answered the questions “Has your previously injured knee had surgery due to redislocation or instability?” and “If yes, can we read your medical record for the surgery report?” These questionnaires were mailed, and completed forms were returned by post. One investigator (M.A.) reviewed electronic medical records for participants who had undergone surgery, and she entered the questionnaire responses into a secure study database.

### Outcome Measures at 10-Year Follow-up

*Primary outcomes:*

— The redislocation rate was classified as early redislocation (≤2 years) or late redislocation (>2 years).
— Whether patients underwent additional patellar stabilizing surgery after redislocation was classified as yes/no.

*Secondary outcomes:*

— Subjective knee function was assessed using the KOOS-Child (with subscales Pain, Symptoms, Activities of Daily Living, Sport/Play, and Quality of Life),^19^ the Kujala Anterior Knee Pain Scale,^14^ and the Tegner Activity Score.^27^
— Anatomic patellar instability risk factors (APIFs) were collected during the original RCT^2^ and were assessed using sagittal and axial MRI views. APIFs were considered positive with the following thresholds: trochlear dysplasia: trochlear depth <3 mm;^21^ lateral patellar tilt ≥20°,^8^ elevated tibial tubercle–trochlear groove (TT-TG) distance ≥15 mm,^25^ patella alta: Caton-Deschamps index ≥1.2,^7^ and Insall-Salvati index >1.3.^10,17^

### Statistical Analysis

Data are presented as mean (range) or count (percentage). Group differences were analyzed using the Pearson chi-square test for categorical data, and due to the small sample size, a nonparametric test (Mann-Whitney *U* test) was used for continuous variables. A 2-sided *P* < .05 was considered statistically significant. Analyses were performed with IBM SPSS Statistics Version 29.

## Results

### Patient Characteristics and Follow-up

**Figure 1.**
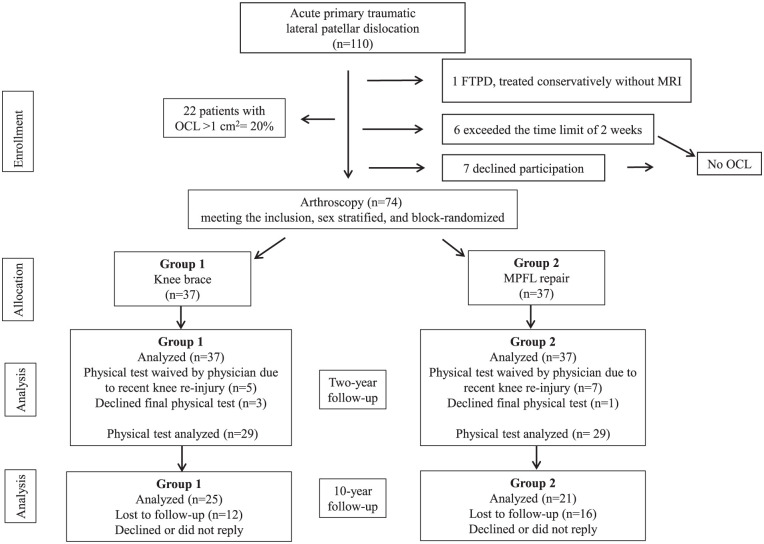
Patient flowchart of individuals in 10-year follow-up of a randomized controlled trial. LPD, lateral patellar dislocation; OCL, osteochondral lesion.

Of the 74 original participants (37 in each intervention group), 46 patients (62%) completed the 10-year follow-up: 25 patients in the KB group and 21 patients in the MPFL repair group (Figure 1). Baseline characteristics were similar between groups, and APIFs were common in both groups and did not differ significantly (Table 1).

**Table 1:** Demographic Characteristics and Anatomic Patellar Instability Risk Factors of Patients Treated With Knee Brace or MPFL Repair After First-Time Traumatic Patellar Dislocation*^*a*^*

	Knee Brace (n = 25)	MPFL Repair (n = 21)	*P*
Demographic characteristic			
Follow-up time, y	10 (9-12)	10 (9-12)	.930*^*b*^*
Age at injury, y	13 (11-15)	14 (12-15)	.168*^*b*^*
Age at follow-up, y	24 (21-26)	24 (22-26)	.337*^*b*^*
Female sex	15 (60)	10 (48)	.401*^*c*^*
Risk factor			
Trochlear depth <3 mm	6 (24)	6 (29)	.725*^*c*^*
Patellar tilt ≥20°	15 (60)	9 (43)	.246*^*c*^*
TT-TG distance ≥15 mm	8 (32)	6 (29)	.801*^*c*^*
Caton-Deschamps index ≥1.2	19 (76)	18 (86)	.408*^*c*^*
Insall-Salvati index >1.3	12 (48)	10 (48)	.979*^*c*^*

a Data are expressed as mean (range) or n (%) of patients. MPFL, medial patellofemoral ligament; TT-TG, tibial tubercle–trochlear groove.

b Mann-Whitney *U* test was used to test homogeneity between the intervention groups.

c Pearson chi-square test was used to test homogeneity between the intervention groups.

### Redislocation Rates and Subsequent Surgery

The overall redislocation rate was higher in the KB group (80%; 20/25) than in the MPFL repair group (62%; 13/21), but the difference was not statistically significant (*P* = .175). Early redislocations (≤2 years) occurred in 52% of the KB group (13/25) versus 29% of the MPFL repair group (6/21) (*P* = .108). Late redislocation rates were similar between groups (28% [7/25] vs 33% [7/21]; *P* = .695).

Among patients who redislocated, subsequent knee surgery was performed in 45% (9/20) of those initially treated with KB and in 54% (7/13) of those in the MPFL repair group. The surgeries were patellar stabilizing procedures in 14 knees, removal of loose bodies in 2 knees, and microfracturing of osteochondral injury in 2 knees. Three of these 16 patients had surgery on the contralateral knee (Appendix Table A1). Patients treated with KB underwent additional surgery significantly earlier (mean 25 months) than those in the MPFL repair group (mean 62 months; *P* = .016) (Table 2).

**Table 2:** Redislocation Rates and Time to Subsequent Knee Surgery in Patients Treated With Knee Brace or MPFL Repair After First-Time Traumatic Patellar Dislocation*^*a*^*

	Knee Brace (n = 25)	MPFL Repair (n = 21)	*P*
Redislocation	20 (80)	13 (62)	.175*^*b*^*
Redislocation within 2 y	13 (52)	6 (29)	.108*^*b*^*
Redislocation after 2 y	7 (28)	7 (33)	.695*^*b*^*
Redislocation without additional knee surgery	11 (44)	6 (29)	.619*^*b*^*
Redislocation with additional knee surgery	9 (36)	7 (33)	.619*^*b*^*
Redislocation with additional knee surgery within 2 y from the first dislocation	6 (24)	0 (0)	.016*^*b*^*
Redislocation with additional knee surgery after 2 y from the first dislocation	3 (12)	7 (33)	.081*^*b*^*
Time to subsequent knee surgery, mo	25 (4-73)	62 (24-142)	.016*^*c*^*

a Data are expressed as n (%) of patients or mean (range). MPFL, medial patellofemoral ligament.

b Pearson chi-square test was used to test homogeneity between the intervention groups.

c Mann-Whitney *U* test was used to test homogeneity between the intervention groups.

### Long-term Subjective Knee Function: KB Versus MPFL Repair

None of the subjective knee function scores differed significantly at 2-year follow-up between participants who completed follow-up and those lost to follow-up, indicating a limited risk of selection bias (Table 3).^14,19,27^

**Table 3:** Subjective Knee Function Scores at 2 Years for Patients Treated With Knee Brace or MPFL Repair After First-Time Traumatic Patellar Dislocation*^*a*^*

	Knee Brace			MPFL Repair		
Subjective Knee Function Measure	Included in RCT at 2 y	Included in 10-y Follow-up		Included in RCT at 2 y	Included in 10-y Follow-up	
	(n = 36)	(n = 25 of 36)	*P* *^*b*^*	(n = 36)	(n = 21 of 36)	*P* *^*b*^*
KOOS-Child Pain	87 (25-100)	87 (25-100)	.971	83 (31-100)	83 (44-100)	≥.999
KOOS-Child Symptoms	87 (43-100)	87 (43-100)	.971	82 (50-100)	83 (54-100)	.696
KOOS-Child ADL	94 (38-100)	94 (38-100)	.759	92 (50-100)	92 (64-100)	.966
KOOS-Child Sport/Play	80 (0-100)	79 (0-100)	.942	70 (11-100)	70 (11-100)	.929
KOOS-Child QOL	73 (17-100)	73 (17-100)	.908	63 (21-100)	66 (21-96)	.708
Kujala Anterior Knee Pain Scale	95 (68-100)*^*c*^*	95 (68-100)*^*d*^*	.932	91 (46-100)*^*c*^*	91 (57-100)*^*d*^*	.920
Tegner Activity Score	4.89 (2-8)*^*e*^*	4.83 (2-8)*^*d*^*	.962	4.53 (2-9)*^*d*^*	4.71 (2-9)	.874

a Data are expressed as mean (range). ADL, Activities of Daily Living; KOOS-Child, Knee injury and Osteoarthritis Outcome Score for children; MPFL, medial patellofemoral ligament; QOL, Quality of Life.

b Mann-Whitney *U* test was used to compare scores at 2 years between the whole group and the subset of patients treated with a knee brace (n = 36 vs 26, respectively) or MPFL repair (n = 36 vs 21) for first-time traumatic patellar dislocation who chose to complete the long-term follow-up.

c Data were unavailable for 3 participants.

d Data were unavailable for 1 participant.

e Data were unavailable for 2 participants.

At the 10-year follow-up, no between-group differences reached statistical significance, but the KB group showed slightly higher mean values on all functional outcomes (Table 4). At 10-year follow-up, Tegner Activity Scores (range, 1-10) were modest in both groups, with mean values of 3.72 (range, 0-7) in the KB group and 3.24 (range, 0-7) in the MPFL repair group, indicating that no patients reported very high activity levels.

**Table 4:** Subjective Knee Function Scores at 10 Years for Patients Treated With Knee Brace or MPFL Repair After First-Time Traumatic Patellar Dislocation*^*a*^*

Subjective Knee Function Measure	Knee Brace (n = 25)	MPFL Repair (n = 21)	*P* *^*b*^*
KOOS-Child Pain	85 (53-100)	74 (3-100)	.231
KOOS-Child Symptoms	82 (46-100)	71 (11-100)	.159
KOOS-Child ADL	96 (75-100)	86 (41-100)	.116
KOOS-Child Sport/Play	75 (36-100)	61 (18-100)	.075
KOOS-Child QOL	69 (29-100)	62 (21-96)	.259
Kujala Anterior Knee Pain Scale	81 (49-100)	77 (47-100)*^*c*^*	.393
Tegner Activity Score	3.72 (1-7)	3.24 (1-7)	.283

a Data are expressed as mean (range). ADL, Activities of Daily Living; KOOS-Child, Knee injury and Osteoarthritis Outcome Score for children; MPFL, medial patellofemoral ligament; QOL, Quality of Life.

b Mann-Whitney *U* test was used to compare scores at 10 years between the patients treated with knee brace and MPFL repair for first-time traumatic patellar dislocation.

c Data were unavailable for 2 participants.

### No Redislocation Versus Redislocation

Subgroup analyses revealed that anatomic patellar instability risk factors were common among both individuals without redislocation (n = 13) and those who experienced 1 or more redislocations (n = 33) (Table 5).

**Table 5:** Anatomic Patellar Instability Risk Factors for Patients Treated With Knee Brace or Medial Patellofemoral Ligament Repair for First-Time Traumatic Patellar Dislocation Stratified by Redislocation Status During Follow-up*^*a*^*

Anatomic Patellar Instability Risk Factor	No Redislocation (n = 13)	Redislocation (n = 33)	*P* *^*b*^*
Trochlear depth <3 mm	5 (38)	7 (21)	.230
Patellar tilt ≥20°	8 (62)	16 (48)	.425
TT-TG distance ≥15 mm	3 (23)	11 (33)	.496
Caton-Deschamps index ≥1.2	9 (69)	28 (85)	.229
Insall-Salvati index >1.3	5 (38)	17 (52)	.425

a Data are expressed as n (%) of patients. TT-TG, tibial tubercle–trochlear groove.

b Pearson chi-square test was used to compare the presence of different anatomic patellar instability risk factors between patients who had no redislocation and those who had a redislocation.

Subgroup analyses suggested better knee function in children who did not experience a redislocation, irrespective of the initial treatment. In the KB group, patients who underwent stabilizing surgery within 2 years reported higher subjective knee scores at 10-year follow-up than those in both groups that received stabilizing surgery after the first 2 years. In contrast, the poorest outcomes in the MPFL repair group were seen in children who redislocated but never proceeded to subsequent knee surgery. Because of the small subgroup sizes, no statistical testing was performed, and the observed differences should be interpreted as exploratory only (Table 6).

**Table 6:** Subjective Knee Function Scores at 10-Year Follow-up Stratified by Treatment, Redislocation Status, and Timing of Additional Surgery After First-Time Traumatic Patellar Dislocation*^*a*^*

	No Redislocation		Redislocation Without Additional Surgery		Redislocation With Additional Surgery Within 2 Years From the First Dislocation		Redislocation With Additional Surgery After 2 Years From the First Dislocation	
Subjective Knee Function Measure	Knee Brace (n = 5)	MPFL Repair (n = 8)	Knee Brace (n = 11)	MPFL Repair (n = 6)	Knee Brace (n = 6)	MPFL Repair (n = 0)	Knee Brace (n = 3)	MPFL Repair (n = 7)
KOOS-Child Pain	96 (91-100)	78 (3-100)	84 (53-97)	70 (41-91)	90 (69-100)	NA	64 (53-72)	72 (3-100)
KOOS-Child Symptom	93 (89-100)	75 (11-100)	86 (54-100)	65 (46-79)	80 (61-96)	NA	57 (46-75)	70 (11-96)
KOOS-Child ADL	99 (95-100)	89 (41-100)	95 (75-100)	83 (45-100)	98 (95-100)	NA	86 (80-95)	87 (41-100)
KOOS-Child Sport/Play	91 (79-100)	66 (25-100)	73 (36-100)	45 (18-82)	77 (46-100)	NA	54 (39-71)	69 (36-100)
KOOS-Child QOL	89 (75-100)	70 (29-96)	62 (29-88)	51 (21-79)	76 (50-96)	NA	50 (38-58)	61 (29-96)
Kujala Anterior Knee Pain Scale	97 (94-100)	85 (56-100)*^*b*^*	75 (49-92)	66 (47-82)	88 (74-100)	NA	65 (54-73)	78 (56-95)*^a^*
Tegner Activity Score	4.20 (2-7)	3.00 (1-7)	3.36 (1-6)	2.67 (1-6)	4.00 (2-7)	NA	3.67 (2-6)	4.00 (2-7)

a Scores are expressed as mean (range). ADL, Activities of Daily Living; KOOS-Child, Knee injury and Osteoarthritis Outcome Score for children; MPFL, medial patellofemoral ligament; NA, not applicable; QOL, Quality of Life.

b Data were unavailable for 1 participant. Statistical testing was not performed because of the small sample size.

As shown in Table 7, KOOS-Child Quality of Life and Kujala scores were significantly higher in the no-redislocation group, whereas KOOS-Child Pain reached borderline significance (*P* = .050), indicating a negative impact of redislocation on long-term outcomes. In contrast, scores for KOOS-Child Symptoms, Sport/Play, and Activities of Daily Living and Tegner activity levels did not differ significantly between the groups. Because each subgroup was small, these patterns should be interpreted with caution.

**Table 7:** Subjective Knee Function Scores at 10-Year Follow-up for Patients Treated With Knee Brace or Medial Patellofemoral Ligament Repair Stratified by Redislocation Status After First-Time Traumatic Patellar Dislocation*^*a*^*

Subjective Knee Function Measure	No Redislocation (n = 13)	Redislocation (n = 33)	*P* *^*b*^*
KOOS-Child Pain	85 (3-100)	78 (3-100)	.050
KOOS-Child Symptoms	82 (11-100)	75 (11-100)	.070
KOOS-Child ADL	93 (41-100)	91 (41-100)	.367
KOOS-Child Sport/play	76 (25-100)	66 (18-100)	.216
KOOS-Child QOL	78 (29-100)	61 (21-96)	.025
Kujala Anterior Knee Pain Scale	90 (56-100)*^*c*^*	75 (47-100)*^*c*^*	.004
Tegner activity score	3.46 (1-7)	3.52 (1-7)	.869

a Scores are expressed as mean (range). ADL, Activities of Daily Living; KOOS-Child, Knee injury and Osteoarthritis Outcome Score for children; QOL, Quality of Life.

b Mann-Whitney *U* test was used to compare scores at 10 years between patients who did not redislocate during the study period and those who redislocated.

c Data were unavailable for 1 participant.

## Discussion

The aim of this 10-year follow-up was to describe the long-term consequences of 2 treatment strategies that were in routine clinical use when these skeletally immature patients were first treated, rather than to promote MPFL repair.

This 10-year follow-up study provides rare long-term data comparing knee bracing versus MPFL repair in pediatric patients with FTPD, followed by a standardized, supervised physical therapy program. The MPFL repair group had a lower 10-year redislocation rate than the KB group. Still, the difference was not statistically significant and did not translate into superior KOOS-Child,^19^ Kujala Anterior Knee Pain Scale score,^14^ or Tegner Activity Scale score.^27^ Irrespective of the initial treatment modality, patients who experienced redislocations demonstrated significantly poorer 10-year outcomes compared with those who maintained joint stability.

### Redislocation Rates and Subsequent Surgery

Although the KB group had more redislocations, similar proportions in both groups eventually required subsequent patellar stabilizing surgery. Notably, patients treated with KB underwent additional surgery significantly earlier than those in the MPFL repair group. These findings suggest that although initial treatment may not affect the overall need for subsequent surgery, it does influence when surgery is performed. These results align with 2 published RCTs comparing nonoperative and operative treatment of adolescents with FTPD. Regalado et al,^23^ whose operative arm did not include MPFL repair or reconstruction, reported a 73% redislocation rate and a 27% reoperation rate at a 6-year follow-up in 15 nonoperatively treated children. Palmu et al^20^ found that after a mean of 14 years, the redislocation rate was 71% (20/28) in nonoperatively treated children and 67% (24/36) in operatively treated patients. However, the surgical group received a mix of procedures.

### Long-term Knee Function

At the 10-year follow-up, functional outcomes remained comparable between the patients treated with KB and those who underwent MPFL repair. The minimal clinically important difference (MCID) has been reported as 10 points for the KOOS-Child domains,^5^ 9 points for the Kujala score, and 0.9 points for the Tegner score.^22^ No between-group difference reached statistical significance. However, assuming the published KOOS-Child MCID value holds for our cohort, patients in the KB group achieved MCID in the subscales of Pain, Symptoms, Activities of Daily Living, and Sport/Play.

These results align with a recent Cochrane review^26^ and a meta-analysis,^29^ both indicating no long-term functional advantage for MPFL repair over structured rehabilitation. In these studies, lower early redislocation rates were reported with surgery. A retrospective case series by Xu et al,^28^ who evaluated nonoperatively treated patients (n = 23) and those treated with MPFL repair (n = 61), with a mean follow-up of 41 months (range, 24-61 months), also showed that although MPFL repair reduced early redislocations, functional outcomes were comparable to those of nonoperative treatment. In our cohort, redislocation was associated with worse KOOS-Child and Kujala scores, whereas Tegner scores did not discriminate clearly between patients with and without redislocation.

Notably, all mean functional scores in the present study were lower at 10-year follow-up compared with the 2-year follow-up, regardless of treatment. At 10 years, the relative advantage of MPFL repair in terms of lower redislocation rates diminished, and functional results were similar in both operative and nonoperative patients. These data suggest that individualized nonoperative treatment, including structured physical therapy, remains a viable first-line option for some patients.

Only 3 children experienced redislocation after initial nonoperative treatment and had delayed surgery for >2 years, and they reported the lowest functional scores compared with those who had stabilizing surgery within 2 years after redislocation. These findings highlight the importance of close follow-up after nonoperative treatment and support considering early surgery if symptoms persist, even without redislocation, in line with recent consensus statements.^4^ Many patients required secondary interventions, which involved MPFL reconstructions, sometimes combined with bony realignment. This underscores the importance of a thorough initial assessment, including imaging and clinical examination, to identify risk factors and address them, when necessary, alongside MPFL reconstruction.

Despite small subgroup sizes and the inability to perform statistical testing, the observed pattern suggests that recurrent instability after nonoperative care and MPFL repair may compromise long-term knee function. Although early repair may help reduce immediate redislocations, recent consensus and meta-analyses favor MPFL reconstruction over repair for better mid-term stability.^4,9,12,16^

### No Redislocation Versus Redislocation

Early surgical stabilization may reduce the functional decline that follows redislocation, whereas delayed or absent revision caused the poorest 10-year scores in this study. This pattern appeared in both the KB and MPFL repair cohorts, indicating that the timing of stabilizing surgery and the choice of surgery are important factors in determining long-term knee function (Table 6). This was also partly seen in the study by Moström et al,^18^ even though the stabilization was performed with dated surgical methods.

Skeletally immature patients with FTPD need to be thoroughly investigated to clarify the extent of predisposing anatomic risk factors. In our 10-year cohort, a high number of risk factors were seen in both groups, but the cohort is too small to find a possible statistical difference between the children who redislocated and the ones who did not.

Individual redislocation risk estimation, patients’ demands, and a check for osteochondral or chondral fragments should be performed to stratify between surgical or nonsurgical treatment and should be carefully discussed with the patient and family. From this study, we can see that delayed surgery or no stabilizing surgery after instability caused the poorest outcome. This supports the recommendation from the ESSKA 2024 formal consensus for management of first-time patellar dislocation, that a strict follow-up is required regardless of first treatment.^4^ The statistically significant difference in KOOS-Child Pain and Quality of Life domains and Kujala scores underlines the clinical value of maintaining patellar stability. In contrast, KOOS-Child Symptoms, Sport/Play, and Activities of Daily Living and Tegner scores did not differ significantly, suggesting that certain functional domains may be less sensitive to redislocation. Taken together, these findings reinforce that sustained surveillance and subsequent surgical stabilization are often indicated in FTPD to preserve function and quality of life. In a 2-year follow-up study, Gurusamy et al^9^ showed that MPFL reconstruction had a better outcome than no stabilizing surgery or MPFL repair when performed with concomitant loose body surgery in FTPD. The MPFL reconstruction group had less recurrent instability and required less secondary surgery and a higher rate of returning to sport.

Given the high recurrence and maintained instability in children in this study, we do not support routine MPFL repair. Neither can we support nonoperative treatment as the gold standard for every child, but when nonoperative treatment is used, a thorough follow-up should be planned.

### Strengths and Limitations

This study provides one of the few RCT datasets comparing one type of surgery with one kind of nonoperative treatment, each followed by recommended physical therapy after FTPD in skeletally immature patients, with a 10-year follow-up, following the patients from adolescence to early adulthood. The use of 3 validated PROMs and the stratification of results by redislocation and subsequent surgical intervention add clinical depth. However, using a more modern, disease-specific PROM, such as the Banff Patellofemoral Instability Instrument (BPII) 2.0,^15^ and objective clinical assessment could have provided additional insight into subjective perception and objective signs of instability 10 years after FTPD.

Several other limitations should be acknowledged. First, although baseline MRI in the original RCT^2^ captured key anatomic risk factors, they were not taken into consideration before the randomization that was performed in the first study. Only 46 of the original 74 participants were available at 10 years, leaving the study underpowered for robust subgroup analyses of different risk factors, and thus the study should be regarded as exploratory. Second, a major limitation is that the 36% loss to follow-up also introduces the possibility of selection bias, even though baseline characteristics were comparable between the 2 groups, and knee function at 2 years was similar between respondents and nonrespondents.

Our findings show that MPFL repair did not provide a clear long-term functional advantage over bracing, whereas the superiority of MPFL reconstruction over repair was supported by external literature rather than by the current data. These long-term outcomes provide a benchmark when counseling patients who were treated with KB or MPFL repair in childhood and when comparing the natural history of these strategies with more modern MPFL reconstruction protocols.

## Conclusion

This study offers valuable insights into the long-term outcomes after FTPD, comparing treatment with a soft patellar stabilizing brace versus MPFL repair followed by structured physical therapy. The long-term redislocation rate in FTPD for patients with MPFL repair was nearly as high as for patients treated nonoperatively. Subjective knee function was comparable between the 2 treatments. Given the high rate of recurring instability in children, we do not support routine MPFL repair for FTPD, nor do we support nonoperative treatment as the gold standard for every child. But when nonoperative treatment is used, a structured follow-up over time, including disease-specific PROMs and clinical examination, is mandatory to identify the need for subsequent surgery.

## Appendix

**Supplement Table 1:** Subsequent Knee Surgical Procedures during follow-up.

Patient Number	Initial treatment	Knee	Year	Time to surgery from index dislocation, months	Procedures performed
1	Knee brace	Index	2012	28	Free-body extirpation
1	Knee brace	Index	2014	50	MPFL-R; lateral release Chondral shaving + microfracture
2	Knee brace	Index	2012	32	VMO advancement +/- medial raphy; modified Roux–Goldthwait
2	Knee brace	Contra	2017	N/A	MPFL-R; trochleoplasty; TTD/M; lateral lengthening
3	Knee brace	Index	2011	18	VMO advancement +/- medial raphy; modified Roux–Goldthwait
4	Knee brace	Index	2010	4	VMO advancement +/- medial raphy; modified Roux–Goldthwait
5	Knee brace	Index	2012	18	MPFL-R + modified Roux–Goldthwait
6	Knee brace	Index	2012	19	VMO advancement +/- medial raphy; modified Roux–Goldthwait
7	Knee brace	Index	2012	8	MPFL-R + modified Roux–Goldthwait
8	Knee brace	Index	2018	73	MPFL-R; trochleoplasty; TTD/M; lateral lengthening
9	Knee brace	Index	2013	23	MPFL-R + TTD/M; lateral release
9	Knee brace	Contra	2022	N/A	MPFL-R; trochleoplasty; TTD/M; lateral lengthening
10	MPFL repair	Index	2021	142	MPFL-R; trochleoplasty; TTD/M; lateral lengthening
11	MPFL repair	Index	2013	34	Chondral shaving + microfracture; lateral release
12	MPFL repair	Index	2013	27	MPFL-R + TTM
12	MPFL repair	Contra	2014	N/A	MPFL-R + TTD; lateral release
13	MPFL repair	Index	2018	85	MPFL-R + trochleoplasty
14	MPFL repair	Index	2017	60	MPFL-R
15	MPFL repair	Index	2014	24	MPFL-R + TTD/M; lateral release
16	MPFL repair	Index	2016	63	Free-body extirpation

Index, initially dislocated knee; Contra, contralateral knee; N/A, Not Applicable; MPFL-R, medial patellofemoral ligament reconstruction; TTD/M, tibial-tubercle distalization/medialization; TTM, tibial-tubercle medialization; VMO, vastus medialis obliquus.

Surgeries after 2013 were mainly performed in other hospitals due to the patient's age (over 15 years of age).
